# Supplementary material for: Sources of COVID-19-Related Information in People with Various Levels of Risk Perception and Preventive Behaviors in Taiwan: A Latent Profile Analysis
Source: Int J Environ Res Public Health. 2021 Feb 21;18(4):2091. doi: 10.3390/ijerph18042091 (PMC7924873; doi:10.3390/ijerph18042091)
Supplement: Supplementary file 1 [file ijerph-18-02091-s001.pdf]

Table S1. Measures used in this study.

Measures	Items	Response scale
Risk perception of COVID-19	Item 1: If you were to develop flu-like symptoms tomorrow, would you worry?	1 = not at all worried, 2 = worried less than normal, 3 = about the same, 4 = worried more than normal, 5 = extremely worried
	Item 2: In the past one week, have you ever worried about catching COVID-19?	1 = no, never think about it, 2 = think about it but it didn't worry me, 3 = worried me a bit, 4 = worried me a lot, 5 = worried about it all the time
	Item 3: Please rate the current level of your worry towards COVID-19:	Score ranged from 1-10 (1 = very mild, 10 = very severe)
	Item 4: How likely do you think it is that you will contract COVID-19 over the next 1 month?	1 = never, 2 = very unlikely, 3 = unlikely, 4 = evens, 5 = likely, 6 = very likely, 7 = certain
	Item 5: What do you think are your chances of getting COVID-19 over the next 1 month compared to others outside your family?	1= not at all, 2 = much less, 3 = less, 4 = evens, 5 = more, 6 = much more, 7 = certain
Adoption of protective behaviors	In the past week, did you 1) avoid going to crowded places, 2) wash your hands more often, and 3) wear a mask more often?	0 = no, 1 = yes, but not due to COVID-19, 2 = yes, due to COVID-19
Information sources	Do you obtain COVID-19-related information from 1) Internet media (e.g., Facebook, Twitter, blogs, and Internet news); 2) friends; 3) traditional media (e.g., newspapers, television, and radio broadcasting); 4) academic courses (e.g., online or in-person formal courses lectured by experts); 5) medical staff in health care institutions; 6) coworkers, and 7) family members?	0 = never, 1 = sometimes, 2 = always
